# The effects of iron oxide incorporation on the chondrogenic potential of three human cell types

**DOI:** 10.1002/term.544

**Published:** 2012-03-07

**Authors:** Sushmita Saha, Xuebin B Yang, Steven Tanner, Stephen Curran, David Wood, Jennifer Kirkham

**Affiliations:** 1 Biomaterials and Tissue Engineering Group, Leeds Dental Institute, University of LeedsUK; 2 Medical Physics, Faculty of Medicine & Health, University of LeedsUK; 3 Smith & Nephew Research CentreYork Science Park, UK; 4 Biomineralisation Group, Leeds Dental Institute, University of LeedsUK; 5 NIHR Musculoskeletal Biomedical Research UnitLeeds, UK

**Keywords:** cell labelling, superparamagnetic iron oxide, chondrogenic potential, HBMSCs, neonatal chondrocytes, adult chondrocytes, *in vitro*

## Abstract

Non-invasive monitoring of living cells *in vivo* provides an important tool in the development of cell-based therapies in cartilage tissue engineering. High-resolution magnetic resonance imaging (MRI) has been used to monitor target cell populations *in vivo*. However, the side-effects on cell function of the labelling reagents, such as superparamagnetic iron oxide (SPIO), are still unclear. This study investigated the effect of SPIO particles on the chondrogenic differentiation of human bone marrow stromal cells (HBMSCs), neonatal and adult chondrocytes *in vitro*. Cells were labelled with SPIO for 24 h and chondrogenesis induced in serum-free medium including TGF*β*3. For labelled/unlabelled cells, viability, morphology and proliferation were determined using CellTracker™ Green and PicoGreen dsDNA assays. The expression of *SOX9*, *COL2A1* and *ACAN* was investigated using qRT–PCR after 2, 7 and 14 days. The results showed that viability was unaffected in all of the cells but cell morphology changed towards a 'stretched' phenotype following SPIO uptake. Cell proliferation was reduced only for labelled neonatal chondrocytes. *SOX9* and *COL2A1* expression decreased at day 2 but not at days 7 and 14 for labelled HBMSCs and adult chondrocytes; *ACAN* expression was unaffected. In contrast, *SOX9* and *COL2A1* expression were unaffected in labelled neonatal chondrocytes but a decrease in *ACAN* expression was seen at day 14. The results suggest that downregulation of chondrogenic genes associated with SPIO labelling is temporary and target cell-dependent. Resovist® can be used to label HBMSCs or mature chondrocytes for MR imaging of cells for cartilage tissue engineering. Copyright © 2012 John Wiley & Sons, Ltd.

## 1. Introduction

Cell therapy is an emerging tool in regenerative medicine. Bioluminescence, radioactive labelling and near-infrared fluorescence are some of the techniques that have been used to determine cell fate after applications of cell-based therapies by tracking homing and migration of the transplanted cells (Thorne *et al*., 2006; Contag, 2006; Hofmann *et al*., 2005; Scarff *et al*., 2006). The presence of a high proton density in the extracellular matrix (ECM) of articular cartilage allows magnetic resonance imaging (MRI) to non-invasively monitor cartilage degeneration and regeneration *in vivo* (Jelicks *et al*., 1993; O'Byrne *et al*., 2003). Use of an iron-containing ‘label’ results in a relative increase in both the magnetic susceptibility effects and in the MR transverse relaxation rate. The consequence is image hypo-intensity in regions corresponding to the location of the labelled cells that can be detected using gradient-echo imaging techniques (O'Byrne *et al*., 2003; Hawrylak *et al*., 1993; Josephson *et al*., 1999; Kircher *et al*., 2003; Frank *et al*., 2003; Dodd *et al*., 1999; Yeh *et al*., 1995; Arbab *et al*., 2004a).

A variety of SPIOs are available for use as MRI contrast agents. Ferumoxides (FE) are a popular dextran-coated SPIO, approved by the US Food and Drug Administration (FDA) for *in vivo* human use (Arbab *et al*., 2004a). Dextran-coated SPIO was first used for hepatic MRI (Stark *et al*., 1988; Weissleder, 1994). Thereafter, a number of studies showed that transfection agents (TAs), such as protamine, poly-l-lysine and lipofectamine, interact electrostatically with the SPIO. These are an effective and stable means to non-covalently bind to DNA and internalize the MR label into the cells (Frank *et al*., 2003; Kostura *et al*., 2004). However, the use of TA at high concentrations is said to be toxic, due to electrostatic binding of TA polycations to voltage-gated ion channels, resulting in transient blockage of ion transport (Rink *et al*., 1994). Thus, the use of SPIO without a TA is held to be a relatively more favourable labelling methodology (Mailander *et al*., 2008). Recently, a number of SPIO particles that do not require the assistance of TAs, such as Resovist® (generic name, ferucarbotran; Schering, Germany) and Feridex®, have been made commercially available (Mailander *et al*., 2008). Resovist comprises 10–12 nm magnetic cores stabilized in a carboxydextran biopolymer, which is anionic under physiological conditions (pH 7.4) (Mailander *et al*., 2008). The overall hydrodynamic diameter of Resovist SPIO particles is 62 nm (measured by photon correlation spectroscopy) and they can increase to a size that is detectable via light microscopy following Prussian blue staining (Mailander *et al*., 2008; Reimer and Balzer, 2003). The carboxydextran coating prevents aggregation and allows for aqueous solubility of the nanoparticles (Reimer and Balzer, 2003). Mailander *et al*., (2008) showed Resovist to label cells more efficiently than Feridex without a TA (Mailander *et al*., 2008). More importantly, Resovist is approved for clinical use in Europe, Japan and Australia (Vogl *et al*., 1996; Lutz *et al*., 2005; Wersebe *et al*., 2006).

Recently, there has been some concern regarding the potential effects of SPIO labelling on cellular chondrogenic differentiation; however, the results so far have been inconsistent (Rink *et al*., 1994; Bulte *et al*., 2004). The aim of this study was to examine the effect of Resovist® on the chondrogenic potential of human bone marrow stromal cells (representing a progenitor cell population), human neonatal chondrocytes (representing an immature cell population) and human adult chondrocytes (representing a mature cell population) when labelled with Resovist *in vitro* and to determine whether the inhibitory effects, if any, are target cell-dependent.

## 2. Materials and methods

Tissue culture flasks and plates were obtained from Nunc™ (Scientific Laboratory Supplies, Nottingham, UK). Passage 1 HBMSCs (obtained from donors aged 38 and 40 years) and adult chondrocytes (obtained from donors aged 35 and 40 years) were obtained from Lonza (Slough, UK). The cells were from haematologically normal donors. Passage 0 neonatal cells were provided by Smith and Nephew (York, UK), with appropriate ethical approval and consent for use in commercial and collaborative research.

### 2.1. Basic and chondro-inductive cell culture

HBMSCs, neonatal and adult chondrocytes were maintained in basal [*α*-MEM with 10% fetal calf serum (FCS)] or chondro-inductive media [serum-free *α*-MEM supplemented with 10 ng/ml TGF-*β*3 (Peprotech, USA), 10^−8^ m dexamethasone, 100 µm ascorbate-2-phosphate, 1 × insulin transferring selenium (ITS) supplement] (Johnstone *et al*., 1998; Malpeli *et al*., 2004). The medium was changed every 3 days and cells were passaged at 70–75% confluency. Passage 4 (P4) cells were used for all of the work described here. For chondro-induction, cells were cultured in chondro-inductive medium for 2, 7 and 14 days.

### 2.2. Magnetic cell labelling

All three cell types were incubated in 0.5 µm Fe/ml Resovist serum-free culture medium at 37°C and 5% CO_2_ for 24 h (Mailander *et al*., 2008). The labelled cells were then washed three times with 1× phosphate-buffered saline (PBS; Sigma, UK) to eliminate extracellular SPIO (Ittrich *et al*., 2007). Control groups for each cell type were cultured under similar conditions but without Resovist.

### 2.3. Detection of intracellular iron content

Prussian blue staining was used to detect the presence of iron oxide in the cells. Briefly, labelled and unlabelled cells were fixed with 10% neutral buffer formaldehyde. The cells were stained for 30 min with aqueous potassium ferrocyanide (10%) mixed in equal parts with 20% aq. hydrogen chloride (Perl's Stain Kit, TCS Biosciences, UK) and counterstained in 0.1% nuclear fast red solution for 5 min after three changes of distilled water. The slides were then mounted in *p*-xylene bis-pyridinium bromide (DPX) and observed under an Olympus BX50 microscope, using SPOT software (version 1.2).

### 2.4. Evaluation of viability and morphology of iron oxide-labelled cells

The cellular viability and morphology for labelled and unlabelled HBMSCs, neonatal and adult chondrocytes were visually evaluated. Briefly, cells were labelled with CellTracker™ Green CMFDA (5-chloromethylfluorescein diacetate, Invitrogen; 50 µg in 5 ml culture medium) for 45 min and washed thoroughly with serum-free medium twice at 45 min intervals (Partridge *et al*., 2002; Green *et al*., 2004; Yang *et al*., 2006). The cells were then imaged under a fluorescent inverted microscope.

### 2.5. PicoGreen® dsDNA quantitation assay

Cells were seeded at 4 × 10^3^ cells/well of each cell type in six-well plates and cultured in basal medium overnight, followed by labelling with or without Resovist (*n =* 6 for both experimental samples and controls), as previously described. After 2, 7 and 14 days of culture in chondrogenic medium, the cells were lysed using 0.1% Triton X-100 and total DNA content was determined using PicoGreen fluorescence reagent, according to the manufacturer's instructions (Green *et al*., 2003; Green *et al*., 2005; Yang *et al*., 2006). Sample fluorescence was measured using a Fluoroskan Ascent 2.6 micro-plate reader (excitation 480 nm, emission 520 nm).

### 2.6. Real-time quantitative reverse-transcription polymerase chain reaction (qRT–PCR)

Resovist®-labelled and unlabelled cells were cultured in T25 flasks for 2, 7 and 14 days in chondrogenic medium (*n =* 3). Total RNA was isolated from the samples using the RNeasy™ mini-kit (Qiagen, UK). RNA (1 µg) was then converted to cDNA using a high-capacity cDNA kit (Applied Biosystems, UK). Real-time RT–PCR was performed (Rotor Gene 6000 Real Time PCR system, Corbett Research, UK). The TaqMan gene expression assay (Applied Biosystems) was used to analyse the expression of chondrogenic markers: Sox9 (*SOX9*), collagen type II (*COL2A1*) and aggrecan (*ACAN*) genes (Grogan *et al*., 2007; Chubinskaya *et al*., 1998; Malladi *et al*., 2006), together with glyceraldehyde-3-phosphate dehydrogenase (*GAPDH)* as the housekeeping gene. Our previous publication showed that *GAPDH* was a reliable housekeeping gene, as its expression levels did not differ between experimental and control samples for the three cell types (Saha *et al*., 2010). The primer details used for these genes can be found in Table 1. The comparative Ct method (2^–*ΔΔ*^Ct) was employed for quantification of gene expression, in which expression levels of target genes in labelled cells are normalized to the unlabelled controls (Jakob *et al*., 2001; Afizah *et al*., 2007; Dyson *et al*., 2007). An expression level < 1.0 in the labelled cells therefore indicates a downregulation of that gene relative to its expression in the unlabelled control (Saha *et al*., 2010).

**Table 1:** Primers used in TaqMan gene expression assays

Gene	Ref. sequence	Assay ID	Exon boundary	Assay location	Amplicon length
*GAPDH*	NM_002046.3	Hs99999905_m1	3–3	154	122
*SOX9*	NM_000346.3	Hs00165814_m1	2–3	1060	102
*COL2A1*	NM_033150.2	Hs00264051_m1	6–7	503	124
*ACAN*	NM_013227.3	Hs00153936_m1	11–12	2644	91

### 2.7. Statistical analysis

Statistical analysis of 2^–*ΔΔ*^Ct values was performed using one-way analysis of variance (ANOVA) with Tukey–Kramer multiple comparisons post-test, using GraphPad Instant Software (GraphPad Software, Inc., San Diego, CA, USA).

## 3. Results

### 3.1. Detection of intracellular iron content

**Figure 1:**
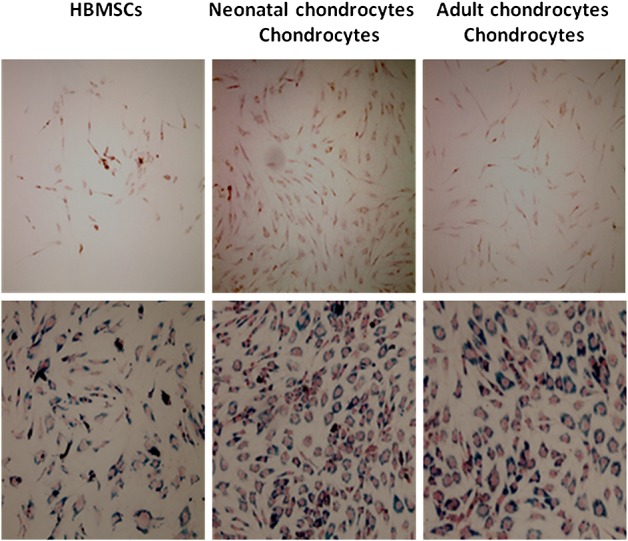
Perl's Prussian blue (PPB) staining of HBMSCs, neonatal and adult chondrocytes. Light micrographs showing: (A–C) unlabelled cells; (D–F) Resovist-labelled cells. Blue stain indicates successful cell labelling with Resovist after 24 h. The unlabelled cells were unstained by PPB and only stained for Congo red. Magnification, ×10

After 24 h of incubation with Resovist (0.5 µm Fe/ml), Perl's Prussian blue staining revealed cytoplasmic uptake of the iron oxide nanoparticles for all three cell types, as indicated by positive blue-stained intracellular vesicles (Figure 1), although the amount of staining varied. Non-labelled cells for all three cell types were unstained by Perl's Prussian blue.

### 3.2. Effect of SPIO labelling on cell viability

**Figure 2:**
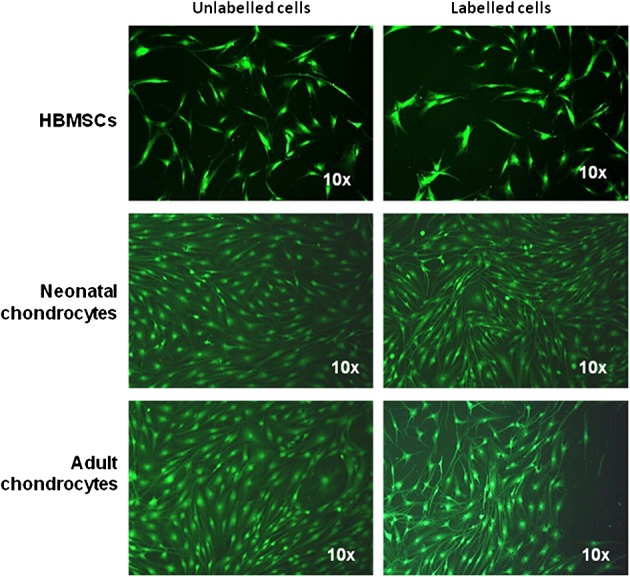
Effect of SPIO labelling on cell morphology. Both labelled and unlabelled cells for each cell type were observed to be viable (stained fluorescent green with CFMDA). However, the morphology of labelled neonatal and adult chondrocytes appeared to be more stretched out and flattened in comparison to the unlabelled samples for each cell type. HBMSCs, human bone marrow stromal cells; NC, neonatal chondrocytes; AC, adult chondrocytes

Resovist labelling had no observable adverse effects on cell viability when compared to non-labelled controls for all three cell types, as evidenced by staining with CMFDA (Figure 2). The fluorescent CMFDA labelling also permitted visualization of cell morphology. This suggested that labelling might be associated with changes to cell morphology, especially in the case of labelled neonatal and adult chondrocyte cell groups, where the cells appeared to be more stretched out and 'stringy' in comparison to the unlabelled cells. However, this preliminary observation would need to be confirmed using quantitative histomorphometric data in future studies.

### 3.3. Effect of SPIO labelling on cell proliferation

**Figure 3:**
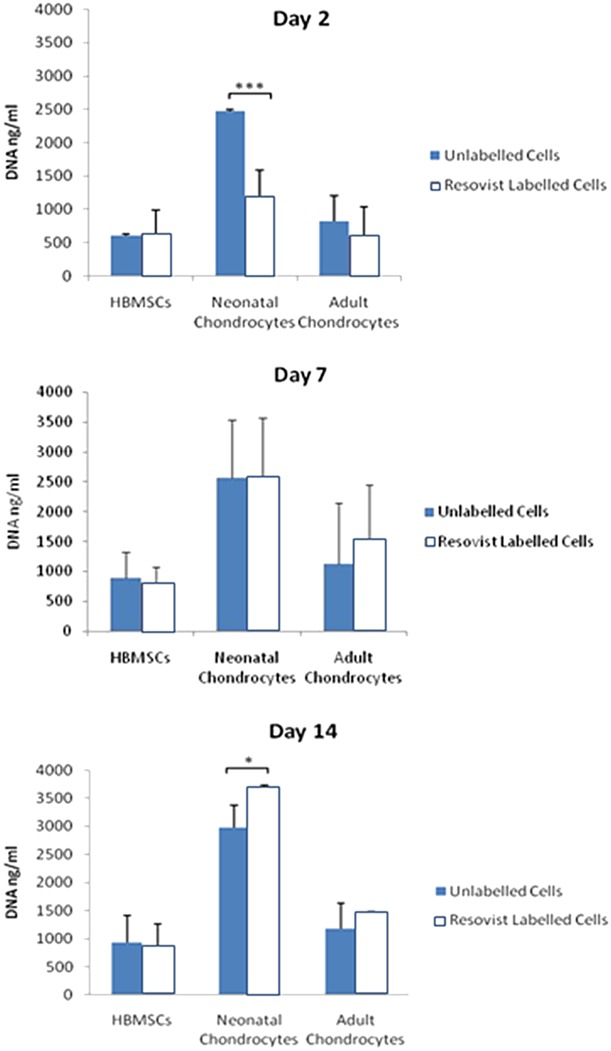
Effect of Resovist labelling on cell proliferation. A significant difference in cell proliferation between labelled and unlabelled cells was observed only in the neonatal chondrocyte group. Results are expressed as mean ± SD (*n =* 3): *p < 0.05; ***p < 0.001

There was no significant difference in cell proliferation between labelled and unlabelled HBMSCs and adult chondrocytes at any of the three time points (Figure 3). However, a highly significant decrease in the proliferation of labelled neonatal chondrocyte cells was observed in comparison to unlabelled control cells after 2 days of culture (*p <* 0.001). After 7 days, there was no significant difference in proliferation between labelled and unlabelled neonatal chondrocytes (*p* > 0.05). After 14 days, labelled neonatal chondrocytes were observed to have a significantly higher proliferation rate than the control cells (*p <* 0.05).

### 3.4. Effect of Resovist labelling on chondrogenesic gene expression

qRT–PCR revealed temporal differences in expression of *SOX9*, *ACAN* and *COL2A1* between labelled and unlabelled cells for all three cell types under chondrogenic culture conditions.

**Figure 4:**
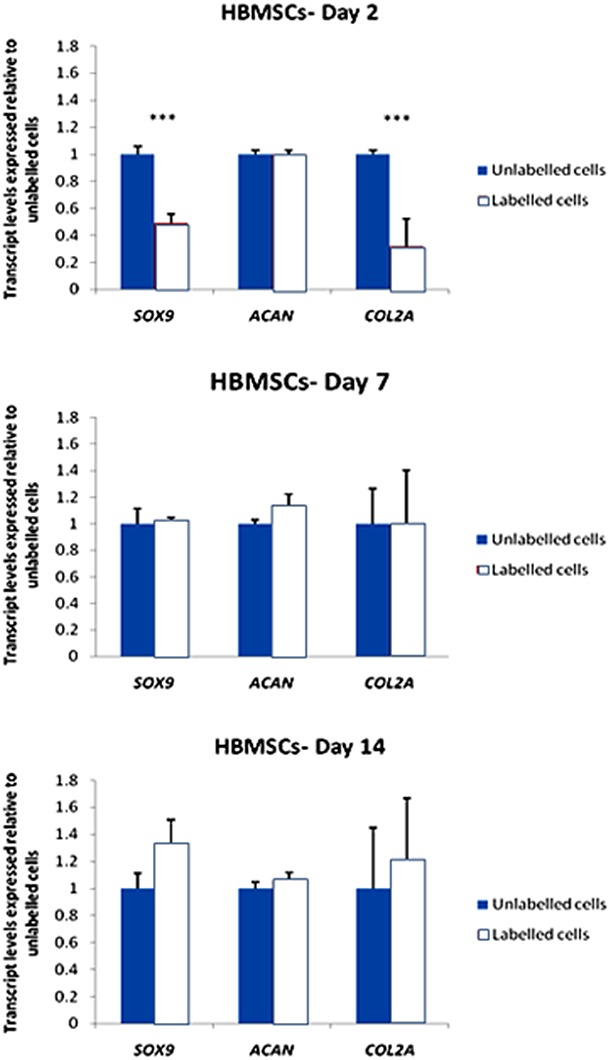
Effect of Resovist labelling on chondrogenic gene expression by HBMSCs. Resovist labelling affected expression levels of *SOX9* and *COL2A1* during the initial stages (day 2) in chondrogenic culture. Gene expression levels were then similar for labelled and unlabelled cells at days 7 and 14. Results are expressed as mean ± SD (*n =* 3): *p < 0.05; **p < 0.01; ***p < 0.001

For HBMSCs, a significant decrease in the expression of *SOX9* and *COL2A1* was observed in the labelled cells after 2 days of culture compared to unlabelled cells (*p <* 0.01), while the expression levels of *ACAN* appeared to be unaffected by the labelling. However, after 7 and 14 days, the expression levels for *SOX9*, *ACAN* and *COL2A1* were not significantly different between labelled and unlabelled cells (Figure 4).

**Figure 5:**
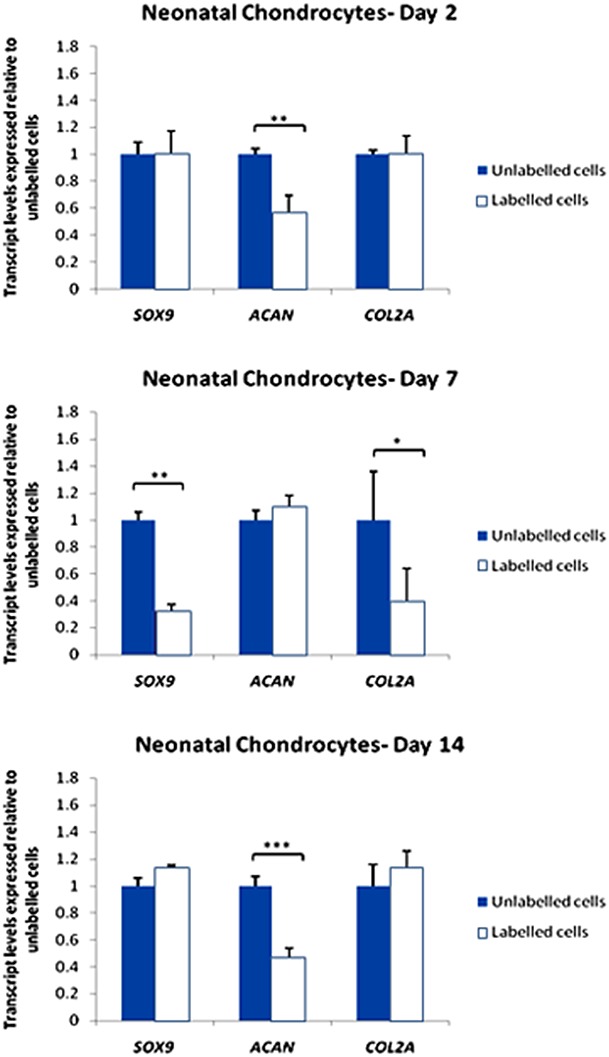
Effect of Resovist labelling on chondrogenic gene expression by neonatal chondrocytes. Resovist labelling did not affect the expression of *SOX9* and *COL2A* at days 2 and 14; a downregulation of the expression levels of these genes was seen at day 7. *ACAN* expression levels were similar between labelled and unlabelled cells at day 7, but a significant decrease in expression was seen at days 2 and 14. Results are expressed as mean ± SD (*n =* 3): *p < 0.05; **p < 0.01; ***p < 0.001

For neonatal chondrocytes, there was a significant (*p <* 0.01) downregulation of *SOX9* and *COL2A1* gene expression in the labelled group compared to the unlabelled group after 7 days of culture in chondrogenic medium, but no significant differences were detected at days 2 and 14 (Figure 5). A. significant decrease in the expression of *ACAN* was observed in the labelled cells after 2 and 14 days of chondrogenic culture compared to unlabelled controls. However, there were no significant differences at day 7 (Figure 5).

**Figure 6:**
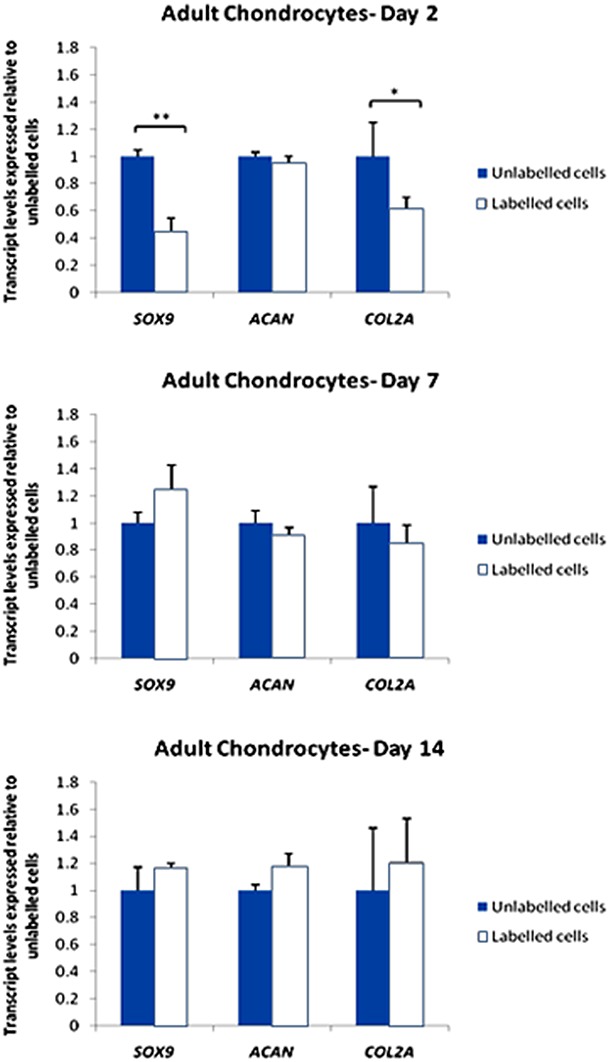
Effect of Resovist labelling on chondrogenic gene expression by adult chondrocytes. Resovist labelling affected expression levels of *SOX9* and *COL2A1* during the initial stages (day 2) of chondrogenic culture only. Gene expression levels were similar for labelled and unlabelled cells at days 7 and 14. No significant differences were seen in *ACAN* expression at any of the time intervals used. Results are expressed as mean ± SD (*n =* 3): *p < 0.05; **p < 0.01; ***p < 0.001

In the case of adult chondrocytes, SPIO labelling resulted in a similar chondrogenic gene marker expression to that of HBMSCs (Figure 6). A statistically significant decrease in expression levels of *SOX9* and *COL2A1* in the labelled group was observed after 2 days of the culture in comparison to the unlabelled group, followed by no significant differences in expression levels of all three genes at days 7 and 14.

## 4. Discussion

SPIO labelling of cells is a popular technique employed as a non-toxic and non-invasive method for monitoring cells and cell therapy using MR imaging (Dodd *et al*., 1999; Ittrich *et al*., 2007; Lewin *et al*., 2000; Hoehn *et al*., 2002). There has been a debate around the labelling of cells with SPIO and its possible effect(s) on cell differentiation (Arbab *et al*., 2004a; Kostura *et al*., 2004; Bulte *et al*., 2004). Our study on the effects of SPIO labelling on chondrogenesis was carried out using three different human cell types and used gene expression as a measure of labelling impact on chondrogenic phenotype, rather than cell staining or the detection of cell surface markers.

Arbab *et al*. (2004) labelled MSCs with ferumoxides, using protamine sulphate as the TA, and showed via histological staining that chondrogenesis was not inhibited (Arbab *et al*., 2004a). In a later report, Arbab *et al*. (2004b) demonstrated similar Alcian blue staining in labelled and unlabelled MSCs, suggesting that ferumoxides did not inhibit chondrogenesis. They also stated that chondrogenic inhibition could arise due to differences in the transfection agent (TA) used or as a result of incomplete washing to remove the iron oxide–TA complexes (Arbab *et al*., 2004b). In contrast, Kostura *et al*. (2004) showed a marked inhibition of chondrogenesis upon labelling MSCs with Feridex coupled to poly-l-lysine as the TA. In their study, chondrogenic inhibition was mediated solely by the SPIO used and was independent of the type of TA employed (Kostura *et al*., 2004). While these studies looked at the possible effects of using an SPIO that required a TA on MSCs, there is still uncertainty about whether the effects of SPIO labelling are dependent on target cell type. In this study, the effects of Resovist without the use of a TA have been investigated on the chondrogenic potential of cells from different sources that might be used for cartilage repair (e.g. HBMSCs, human neonatal chondrocytes and human adult chondrocytes) (Saha *et al*., 2010).

This study confirmed labelling of the three different human cell types after a simple 24 h incubation of the cells with Resovist (0.5 µm SPIO/1 ml culture medium) without the use of a TA. A serum-free labelling protocol was used in this study, as it has been suggested that SPIO nanoparticles form conglomerates with serum which adhere strongly to the cells' surfaces and which cannot be easily removed (Mailander *et al*., 2008). The cell labelling used here was easily detected via MRI and differences between labelled and unlabelled cells (negative staining) in the decay of transverse magnetization (T2) (data not shown).

Our results showed that cell labelling did not appear to alter the viability of any of the three cell types, which is similar to the findings of others (Stark *et al*., 1988; Mailander *et al*., 2008; Bulte *et al*., 2004). Our data also suggested a possible change in cell morphology after labelling. Although this remains to be confirmed in future work, Farrell *et al*. (2008) also reported a phenotypic change in Feridex- labelled MSCs *in vivo*, similar to that seen during endochondral ossification (Farrell *et al*., 2008), and suggested that SPIO labelling inhibited osteogenic but not chondrogenic differentiation of MSCs.

In the present study, Resovist labelling was observed to significantly decrease cell proliferation rates compared to unlabelled control cells only in the neonatal chondrocyte group at the earliest time point used (day 2). At day 14, enhanced proliferation of the labelled cells was detected. It is possible that the enhanced proliferation rate of labelled neonatal chondrocytes at day 14 days may be a compensatory mechanism following the initial downregulation.

Inhibition of chondrogenic gene expression seen after Resovist labelling appeared to be a short-term effect only in the case of HBMSCs and adult chondrocytes. This contradicts previous studies showing the inhibitory effects of SPIO labelling on MSC chondrogenic differentiation (Kostura *et al*., 2004; Bulte *et al*., 2004; Farrell *et al*., 2008). It is possible that the short-term downregulation of chondrogenic genes observed here at day 2 for labelled HBMSCs and adult chondrocytes is related to effects of the label on the membrane-bound transferrin-like protein (MTf), a glycosylphosphatidylinositol anchored protein expressed at high levels by chondrocytes (Oda *et al*., 2003). MTf upon crosslinking with Concanavalin-A, results in a change in cell shape and leads to chondrogenic induction (Oda *et al*., 2003). Expression patterns of MTf are said to parallel that of collagen type II with the onset of chondrogenesis (Nakamasu *et al*., 1999). It has been reported that cellular divisions over time lead to a step wise decrease in the number of SPIO positive cells (Yano *et al*., 2005). Thus, between day 2 of chondrogenesis and day 7, the SPIO concentration should have been diluted considerably, perhaps allowing MTf to resume normal morphology and activity to maintain normal chondrogenic gene marker expression levels. Schafer *et al*., (2009) also observed non inhibition of chondrogenic differentiation upon labelling HBMSCs with Resovist® after 14 days of chondrogenic culture (Schafer *et al*., 2009).

A significant decrease in expression levels of *ACAN* by labelled neonatal chondrocytes was observed in comparison to the control cells at days 2 and 14 in chondrogenic culture. *ACAN* expression levels were seen to recover in the labelled cells at day 7. However *SOX9* and *COL2A1* expression levels were significantly decreased in the labelled cell population at day 7. Our study therefore shows downregulation of key chondrogenic markers after intracellular incorporation of Resovist to be target cell-dependent. The mechanism of downregulation of chondrogenic gene expression by Resovist in neonatal chondrocytes is unknown. A difference in the pattern of temporal expression of chondrogenic gene markers was previously observed between human neonatal chondrocytes and human adult HBMSCs/chondrocytes (Saha *et al*., 2010). It is possible that the Resovist inhibitory effect observed in neonatal chondrocytes at day 14 could be due to intrinsic differences in their signalling pathways compared to adult cells. Different morphological zones in adult/mature cartilage tissue release a variety of bioactive molecules, such as lubricin and cartilage intermediate layer protein (CILP) (Niikura and Reddi, 2007; Lorenzo *et al*., 1998), while numerous proteins are involved in forming ECM trans-activating complexes with SOX9 (SOX5/SOX6,CREB/p300 and c-Maf) (Lefebvre *et al*., 1998; Tsuda *et al*., 2003; Huang *et al*., 2002). These molecules may be capable of reducing/removing any interference that Resovist may have on the intracellular signalling processes.

Human neonatal cartilage is also known to contain two types of chondrocytes (Hwang, 1978). The lack of relevant information regarding the purpose and differences between these two types of chondrocytes makes it difficult to infer how Resovist might affect chondrogenesis in these cells. Further studies on the exact mechanism of chondrogenesis and the different biomolecules involved are warranted, as this study suggested that a 0.5 µm Fe/ml addition of Resovist cannot be used to label human neonatal chondrocytes, due to its effects on the mechanism of chondrogenic differentiation. We note that our use of the comparative Ct method cannot determine threshold levels of gene expression which would impact on the relevant protein expression.

It is possible that the observed short-term effects of labelling are related to a heat shock response to the cellular uptake of Resovist, although this hypothesis remains to be tested. Heat shock protein (HSP) expression can be induced by stress factors, such as heavy metals or reactive oxygen intermediates (Winrow *et al*., 1990; Schett *et al*., 1998), and HSP70 and HSP32 are known to be expressed in chondrocytes under stress (Kaarniranta *et al*., 2001). A high expression of HSP70 in cartilage has been shown to reduce proteoglycan synthesis and induce chondrocyte apoptosis (Sawatzky *et al*., 2005). However, low levels of HSP70 and HSP32 have been shown to be chondroprotective (Sawatzky *et al*., 2005).

The determination of the absolute amounts of SPIO taken up by the cells would have been a useful measure to inform further discussion as to the effects of labelling. The observed differences in the effects of the label on chondrogenesis and cell morphology and proliferation could reflect dissimilar amounts of the iron oxide label taken up within the different cell types.

## 5. Conclusion

This study observed that Resovist at 0.5 µm Fe/ml was incorporated by HBMSCs, neonatal and adult chondrocytes without affecting cell viability. We have also demonstrated, for the first time, that downregulation of chondrogenic gene expression with Resovist is apparently only a transient effect that is target cell-dependent. These results are noteworthy, as Resovist could be used at this concentration as a useful tool for non-invasive monitoring of stem cells and mature chondrocytes *in vitro* and *in vivo* for cartilage repair/regeneration.
